# A functional and transcriptomic analysis of NET1 bioactivity in gastric cancer

**DOI:** 10.1186/1471-2407-11-50

**Published:** 2011-02-01

**Authors:** Gayle Bennett, Denise Sadlier, Peter P Doran, Padraic MacMathuna, David W Murray

**Affiliations:** 1 Gastrointestinal Unit; Mater Misericordiae University Hospital, Dublin 7, Ireland; 2 UCD Clinical Research Centre, UCD School of Medicine and Medical Sciences, Misericordiae University Hospital, Dublin 7, Ireland

## Abstract

**Background:**

NET1, a RhoA guanine exchange factor, is up-regulated in gastric cancer (GC) tissue and drives the invasive phenotype of this disease. In this study, we aimed to determine the role of NET1 in GC by monitoring the proliferation, motility and invasion of GC cells in which NET1 has been stably knocked down. Additionally, we aimed to determine NET1-dependent transcriptomic events that occur in GC.

**Methods:**

An in vitro model of stable knockdown of NET1 was achieved in AGS human gastric adenocarcinoma cells via lentiviral mediated transduction of short-hairpin (sh) RNA targeting NET1. Knockdown was assessed using quantitative PCR. Cell proliferation was assessed using an MTS assay and cell migration was assessed using a wound healing scratch assay. Cell invasion was assessed using a transwell matrigel invasion assay. Gene expression profiles were examined using affymetrix oligonucleotide U133A expression arrays. A student's t test was used to determine changes of statistical significance.

**Results:**

GC cells were transduced with NET1 shRNA resulting in a 97% reduction in NET1 mRNA (p < 0.0001). NET1 knockdown significantly reduced the invasion and migration of GC cells by 94% (p < 0.05) and 24% (p < 0.001) respectively, while cell proliferation was not significantly altered following NET1 knockdown. Microarray analysis was performed on non-target and knockdown cell lines, treated with and without 10 μM lysophosphatidic acid (LPA) allowing us to identify NET1-dependent, LPA-dependent and NET1-mediated LPA-induced gene transcription. Differential gene expression was confirmed by quantitative PCR. Shortlisted NET1-dependent genes included STAT1, TSPAN1, TGFBi and CCL5 all of which were downregulatd upon NET1 downregulation. Shortlisted LPA-dependent genes included EGFR and PPARD where EGFR was upregulated and PPARD was downregulated upon LPA stimulation. Shortlisted NET1 and LPA dependent genes included IGFR1 and PIP5K3. These LPA induced genes were downregulated in NET1 knockdown cells.

**Conclusions:**

NET1 plays an important role in GC cell migration and invasion, key aspects of GC progression. Furthermore, the gene expression profile further elucidates the molecular mechanisms underpinning NET1-mediated aggressive GC cell behaviour.

## Background

Gastric Cancer (GC) is a significant oncological "problem" as it is characterised by a high metastatic potential and a dismal 5-year survival rate of 20%. The disease often presents at an advanced stage, often with lymph node or distant metastases, and patients therefore generally have a poor outcome [1,2]. There is a poor understanding of the natural history of the disease and despite advances in surgery, surgical cure is rare. Conventional chemotherapeutic approaches to dealing with GC are limited and often ineffective. Recent work has identified molecular markers may correlate with disease recurrence following surgery [3,4] and with response to chemotherapy [5]. There is however, a real need to further understand the biology of this disease in order to develop effective targeted therapies. Novel mediators of the disease process, and novel interactions between existing and established mediators, have been identified by our group [6,7]. We propose that inhibition of the molecular mechanisms we have defined may lead to improved therapies for GC patients.

We have shown that NET1, a guanine exchange factor (GEF) for RhoA, is up-regulated in GC tissue and drives its aggressive phenotype. The GEF family of proteins activate GTPases such as RhoA, leading to downstream signalling. NET1 is upregulated by Lysophosphatidic Acid (LPA), a known activator of RhoA, and NET1 drives cancer cell migration, invasion and cytoskeletal actin organisation [7]. In order to further examine the role of NET1 in the disease setting, we created a unique Human Caucasian gastric adenocarcinoma (AGS) cell line with stable knockdown of NET1. This model was used to investigate the role of NET1 in cell proliferation, migration and invasion assays but also to fully identify the downstream transcriptomic events, thereby allowing a unique insight into the genes of importance in GC cell invasion.

Many GC microarray studies have focussed on the identification of genes dysregulated in GC tissue in comparison to normal gastric tissue with the aim of developing an "expression profile" for GC cells [8-12], Hasegawa et al have proposed a 12 gene signature that correlates with lymph node involvement in GC [8]. Gene expression profiles generated by microarray have been used to develop prognostic scores, which when coupled with clinical parameters, have proven useful in predicting aggressive GCs and ultimately, outcome [13]. More recently microarray technology has been employed in the determining survival after resection [14], response to chemotherapy [15,16] and changes in gastric mucosa caused by Helicobacter pylori [17-19]. In this study, using our novel "NET1 knockdown" cell line, microarrays were used to identify NET1 dependent transcriptomic events. We hypothesise that the gene expression profile observed supports the putative pathogenic role of NET1 in GC migration and invasion.

## Methods

### Cell Culture and Short hairpin RNA (shRNA) Transduction

Human Caucasian gastric adenocarcinoma cells (AGS cells) were purchased from the European Collection of Cell Cultures (ECACC) and cultured in Hams F12 medium (Sigma Aldrich), supplemented with 10% fetal bovine serum (FBS), L-glutamine and Penicillin-Streptomycin (Sigma Aldrich), as per ECACC recommendations and incubated at 37°C and 5%CO_2_.

MISSION™ shRNA Lentiviral Transduction Particles (Sigma Aldrich) were used to achieve stable NET1 knockdown. 5 NET1 specific shRNA constructs (named constructs 63, 64, 65, 66 and 67), and one "non target" construct were transduced separately into AGS cells. The "non target" construct contained a short hairpin RNA sequence which did not code for any known human gene, and acted as a control. Briefly, AGS cells were incubated with the NET1-specific shRNA lentiviral particles at a ratio of 2 particles to 1 cell, in the presence of hexadimethrine bromide to improve transduction efficiency. "Non target" shRNA lentiviral particles were used to control for the effects of the transduction process itself. Successfully transduced cells included a puromycin resistance tag, and these cells were selected using 1 μg/ml puromycin. NET1 knockdown was confirmed using quantitative RTPCR and western blot analysis. For the remainder of this study, three distinct cell types were used; NET1 wild type cells (AGS cells which had not been transduced), NET1 knockdown cells (AGS cells transduced with NET1 targeting shRNA) and non-target shRNA cells (transduced with scrambled shRNA which did not target any gene).

### RNA extraction and PCR

RNA extraction by Trizol™, (Sigma Aldrich, Ireland) and reverse transcription were performed as previously described [6]. PCR was performed using Quantitect™ Sybr Green PCR kit (Qiagen™) according to the manufactures instructions. Briefly, 1 μl cDNA was mixed with 6.25 μl of Sybr Green, 4.25 μl DNase free water, and 0.5 μl of forward and reverse primer. The reaction was carried out using a Rotor Gene™ 3000 system. All experiments were carried out in duplicate. β-actin mRNA expression was used to normalize and compare expression values for genes of interest.

### Cell Proliferation Assay

Cell proliferation was assessed by adding 100 μl containing 1 × 10^4 ^cells to three wells of a 96 well plate. Following incubation for 24 hr in serum starved conditions, with or without 10 μM LPA, 20 μl of MTS reagent was added, and following 2 hr incubation at 37°C, 5%CO_2_, absorbance was read at 490 nm using a microplate reader. Three wells containing no cells and MTS only, were used as a blank. All analysis was repeated in triplicate - i.e. on three separate occasions.

### Cell migration

Migration was assessed by wound healing scratch assay as previously described [7]. NET1 knockdown cells (clone 65b), control non target cells and wild type AGS cells were seeded in duplicate wells of a six well plate and grown to 100% confluence. A vertical wound was created in the cell monolayer in each well using a sterile P10 micropipette tip. The wells were then washed with 1 ml of growth medium, which was removed and replaced with 3 mls of serum free growth medium.

The first image of each scratch was acquired at time zero through a phase contrast microscope at 10× magnification. The 6 well plate was then incubated at 37°C, 5% CO_2 _for 14 hours. At 14 hours, each scratch was examined and photographed at the same location. The plate was then returned to the incubator for a total of 24 hours, then removed and photographed again at the same reference point for each scratch. The images acquired for all 3 cell types were compared. The area of the scratch on the 0 hour image was marked by outlining the area that was free from cells, forming a rectangle that was subsequently superimposed on the 14 hour and 24 hour images. The total number of cells migrating into the marked area at 14 and 24 hours were counted. All analysis was repeated in triplicate.

### Invasion assay

Biocoat matrigel invasion chambers were used to compare the effect of NET1 knockdown on in vitro invasion of AGS cells as previously described [6]. Briefly, 500 μl of serum free media containing 1 × 10^5 ^cells of each type - knockdown cells (clone 65b), control non target cells, wild type AGS cells was added into the upper chambers in duplicate and allowed to invade into a lower chamber containing Hams F12 media with 20% FBS by incubating for 24 hr at 5% CO_2 _37°C. Invasive cells were fixed, stained and counted. All experiments were performed in triplicate.

### Microarray Analysis

As described, AGS cells underwent short hairpin mediated NET1 knockdown using 5 different shRNA constructs targeting NET1. A scrambled non-target shRNA was also used as a control. All transduced cells were maintained separately according to the short hairpin RNA construct used for transduction. Biological replicates were made for each construct (five NET1 knockdown cell lines plus one non target control cell line), producing 12 new GC cell lines. Three of those novel cells lines were chosen for further investigation using microarray: Control non target (NT) cells, NET1 knockdown cells "63", and "65". Cells were cultured in duplicate (e.g 63A and 63B) from the time of initial transduction (biological replicates). Prior to mRNA extraction using the Trizol™ method, all three chosen cell lines (63, 65 and non target) were stimulated by 10 μM lyspohosphatidic acid (LPA) a known activator of RhoA [7]. mRNA was therefore available from all 3 cell types in their resting and stimulated states. In total, mRNA was extracted from 12 distinct cell populations.

Prior to hybridisation, RNA quality was determined using Agilent 2100™ bioanalyser. The quality of RNA was determined by its RNA integrity number (RIN) which is an estimate of the integrity of total RNA samples. RIN values range from 10 (intact RNA) to 1 (totally degraded) [20]. All 12 RNA samples were intact and of sufficient quality to progress to the test chip stage (data not shown).

All 12 samples were successfully arrayed. Image files (.cel) were obtained through Affymetrix GeneChip software (MAS5) and were analysed using robust multichip analysis (RMA) express software. Each array was normalised individually, followed by normalisation across all arrays to allow comparison. Only those probe sets with an RMA value of greater than 5.0 were used for further analysis [21,22]. The experimental data for each group was averaged and the change in expression was calculated by comparison of the signal log ratio (SLR) from each sample group vs its control group. A mean signal log ratio (SLR) of 0.6 or greater (equivalent to a 1.5 or greater fold change in expression) as well as a students t test output of < 0.005 were used to identify significant differential expression.

Cluster analysis of the RMA normalised data was used to group genes with similar patterns of expression, as described in Eisen et al 1998 [23]. Correspondence analysis was used to identify major trends in datasets [24,25]. DAVID EASE online tools were used to functionally classify differentially expressed genes. The expression of short listed genes was confirmed using RTPCR as described above.

### Statistical analysis

Data are expressed as mean +/- standard deviation from triplicate experimental replicates and were analysed with Microsoft Excel using Student's t-test with significance defined as p < 0.05

## Results

### Validation of NET1 knockdown

**Figure 1:**
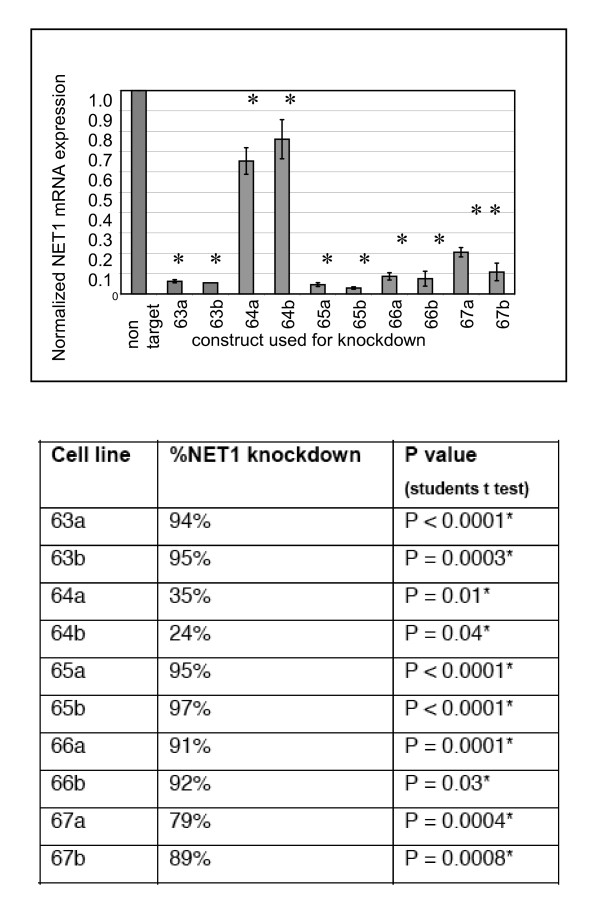
**Confirmation of NET1 knockdown**. Figure 1A; NET1 mRNA expression in AGS gastric cancer cells following transduction with non-targeting scrambled shRNA (control cells) or separate NET1 targeting shRNA molecules. Five separate NET1 targeting shRNA molecules were transduced (denoted; 63, 64. 65. 66 & 67) in duplicate (a & b). All values are expressed relevant to 'non target' control cells, given a value of 1. *p < 0.05 Figure 1B The percentage NET1 mRNA knockdown achieved in AGS cells in comparison with control cells transfected with non-targetting shRNA as determined by RT PCR. Non target cells were given a value of 1 (100% NET1 expression) and all other values were compared to the control sample. *P < 0.05

Following transduction, NET1 knockdown was confirmed by Reverse Transcriptase RT PCR by comparing NET1 mRNA expression levels in cells transduced with NET1 targeting shRNA, versus control cells (transduced with non targeting shRNA) (Figure 1A). NET1 mRNA expression was reduced in all cell lines when compared to control non targeting control cells (P < 0.05). Exact levels of NET1 suppression and p values, indicating significance, are shown in Figure 1B. Clone 65b, which showed the greatest reduction in NET1 mRNA expression was used for the functional studies herein.

### Effect of NET1 knockdown on GC Proliferation in vitro

Following confirmation of knockdown, the proliferative capacities of cells were compared using an MTS assay. There was no significant difference between the proliferation of AGS (clone 65b) cells transduced with NET1 targeting shRNA versus control cells transduced with non-targeting shRNA. Furthermore, transduction with either non-targeting or NET1-specific shRNA had no significant effect on AGS GC cell proliferation compared to wild type non-transduced cells (Data not shown).

### Effect of NET1 knockdown on GC cell migration in vitro

**Figure 2:**
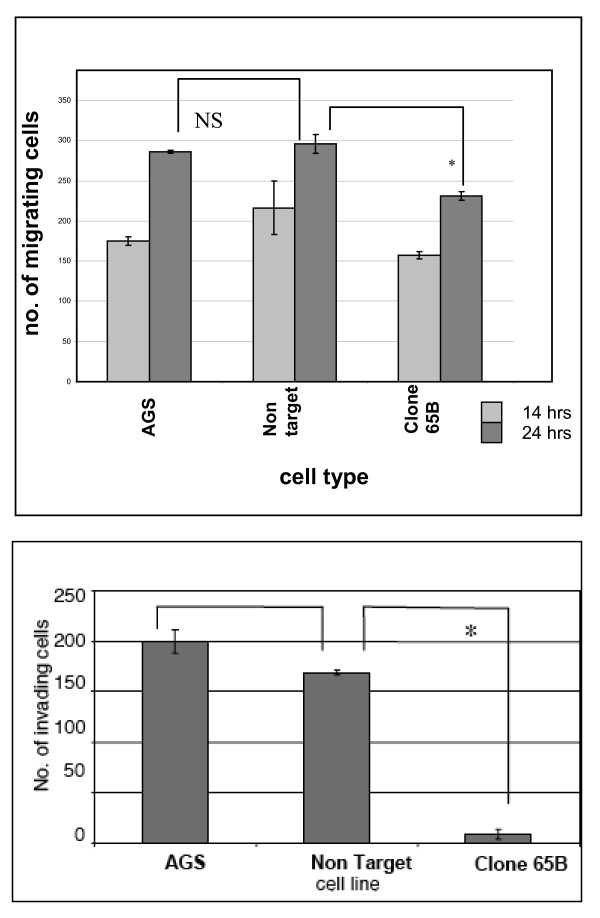
**The role of NET1 in gastric cancer cell migration and invasion**. Figure 2A. The effect of NET1 knockdown on AGS gastric cancer cell migration *in vitro*. Cell migration is expressed as numbers of migrating (i) AGS wild type cells, (ii) control cells tranduced with non targeting shRNA (NT) and (iii) NET1 knockdown cells transduced with NET1 targeting shRNA (65b) at 14 and 24 hr are represented. *p < 0.05. Error bars represent standard deviation of triplicate experiments. Figure 2B: The *in vitro *invasion of AGS, NT and NET1 knockdown cell line (65b). Invasion was expressed as number of invading cells per 10X field. Error bars represent standard deviation of triplicate experiments. *p < 0.0005

Following knockdown, we compared the migration of (i) wild type non-transduced AGS cells (ii) non-targeting scrambled shRNA transduced control cells and (iii) NET1 knockdown cells transduced with NET1 targeting shRNA (clone 65b). This was done using a scratch assay. At 14 hours, migration was not significantly different between AGS wild type non transduced cells and control cells transduced with non targeting scrambled shRNA, as shown in Figure 2A. At 24 hours the migration of NET1 knockdown cells (clone 65b) was significantly reduced compared to control cells transduced with non targeting shRNA and to wild type cells (231 vs 296 vs 285 cells) P < 0.05 (Figure 2A).

### Effect of NET1 knockdown on GC cell invasion in vitro

Following knockdown, cell invasion was assessed using trans-well matrigel invasion inserts. Following NET1 knockdown a 91% reduction in the invasion of AGS cells (clone 65b) was observed, in comparison with the invasion of cells transduced with non-targeting shRNA (p < 0.005) (Figure 2B). The invasion of control cells transduced with non targeting shRNA, which had unchanged levels of NET1, was not significantly different from the wild type non-transduced AGS cells.

### Transcriptomic Effect of NET1 knockdown

**Figure 3:**
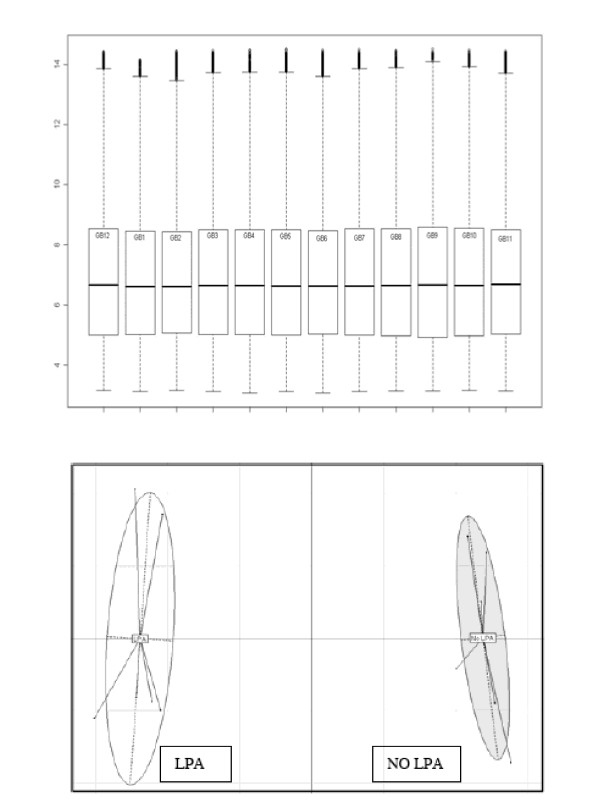
**Data output from transcriptomic analyses**. Figure 3A: Box plots following normalisation of gene array data sets. This Figure shows the spread of data for each individual array. Figure 3B: Correspondence analysis of gene array data from cells treated with or without LPA.

For array comparisons, a standard error of the mean was calculated for each dataset as shown in Figure 3A. A standard error of the mean of ≤ 0.5 was taken as evidence of sufficient similarity between arrays to allow their inclusion for further analysis. As shown in Figure 3A, all samples showed a similar range of distribution and were therefore deemed comparable.

Using correspondence analysis, the arrays separated into two distinct groups reflecting the presence or absence of LPA stimulation by 10 μM LPA (Figure 3B).

**Figure 4:**
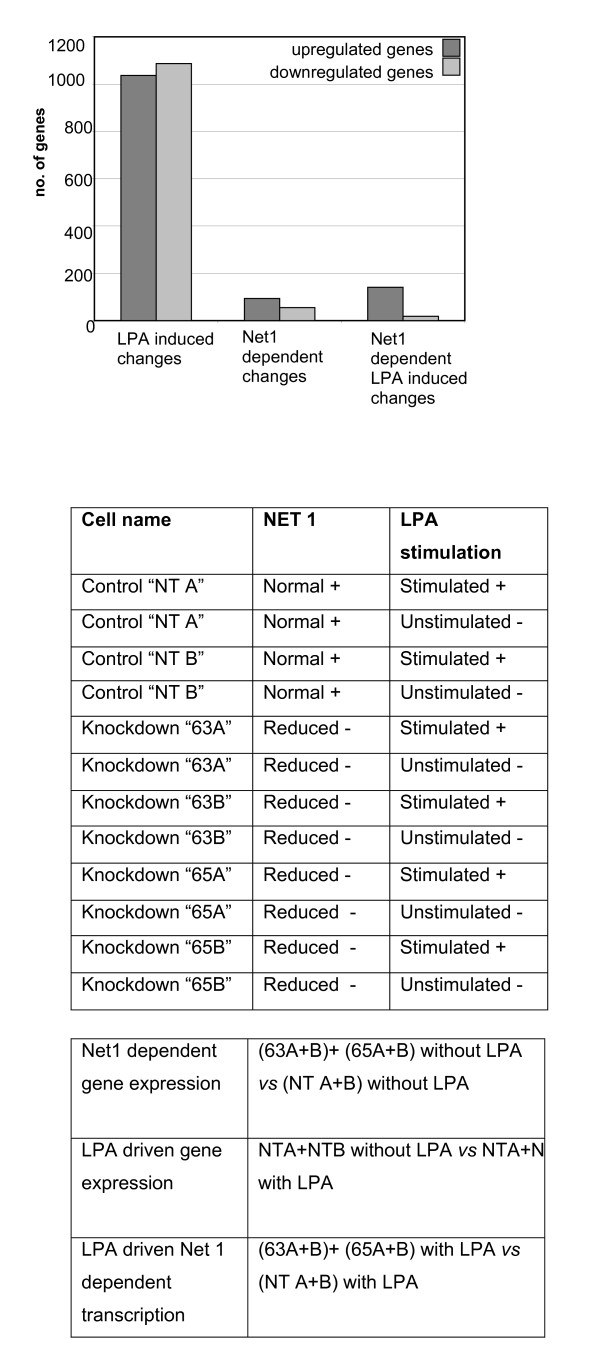
**Data output from transcriptomic analyses and outline of experimental design**. Figure 4A: Global transcriptomic response in each array experiment displaying the number of up and down regulated genes in each array comparison (upper panel). 4B: The unique cell lines from which RNA was extracted for microarray analysis. RNA was extracted from each of the 3 experimental cell lines, control 'non target' cells and knockdown 63 and 65, in duplicate, from cells in resting and stimulated (10 μM LPA) states(center panel). 4C: Microarray data comparisons to determine (1) NET1 dependent transcription, (2) LPA dependent transcriptionand (3) NET1 mediated LPA dependent genetic events (lower panel).

We shortlisted genes with 1.5 fold change in expression and a p value less than 0.05. Three separate comparisons were made using the array data. NET1 dependent gene changes were identified by comparing NET1 knockdown with control (transduced with non targeting shRNA) samples. In this group, 148 genes were significantly dysregulated (93 upregulated, 55 downregulated) (Figure 4A).

To determine LPA driven gene changes, we compared the gene expression profiles of cells transduced with non-targeting scrambled shRNA treated with and without LPA and observed significant dysregulation of > 2000 genes, with approximately equal numbers of genes up- (1087) and downregulated (1037). (Figure 4A).

Finally, we identified NET1-dependent LPA-driven gene expression events by comparison of the expression profiles of (i) LPA-stimulated control cells transduced with scrambled shRNA and (ii) LPA-stimulated knockdown cells transduced with NET1-targeting shRNA. We observed 159 differentially genes expressed with 18 genes significantly downregulated. (Figure 4A).

**Figure 5:**
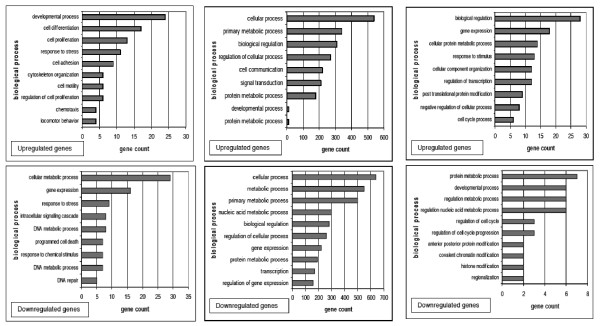
**Functional classification for differentially expressed genes**. Figure 5A: Functional annotation of NET1 dependent upregulated (upper panel) and downregulated (lower panel) genes. Figure 5B: Functional annotation of LPA dependent upregulated (upper panel) and downregulated (lower panel) genes. Figure 5C: Functional annotation of NET1 and LPA dependent upregulated (upper panel) and downregulated (lower panel) genes. Total gene count is represented on the Y axis

For each of the three categories, a list of differentially expressed genes was compiled. These lists were divided into upregulated and downregulated genes for each category. Using DAVID (Database for Annotation, Visualisation and Integrated Discovery) online software, the functional categories of the genes were assigned. (Figure 5).

With high levels of NET1, upregulation of genes involved in cell proliferation, adhesion, cell motility, chemotaxis, and cytoskeletal organisation was demonstrated (Figure 5A upper panel). In contrast, higher levels of NET1 were associated with downregulation of genes involved in regulation of apoptosis, cell death and DNA repair. (Figure 5A lower panel).

The LPA transcriptome demonstrated differential expression of over 2000 genes, the most abundant functional families are displayed in Figure 5B.

Using a combination of the above comparisons we identified gene changes that are driven by LPA but also dependent on NET1 (Figure 5C). Genes involved in metabolic processes, cell cycle regulation, transcription and cell growth were upregulated. (Figure 5C upper panel). Relatively few downregulated genes were detected, those that were related primarily to metabolism. (Figure 5C lower panel).

### Confirmation of Microarray analysis by RT PCR

**Figure 6:**
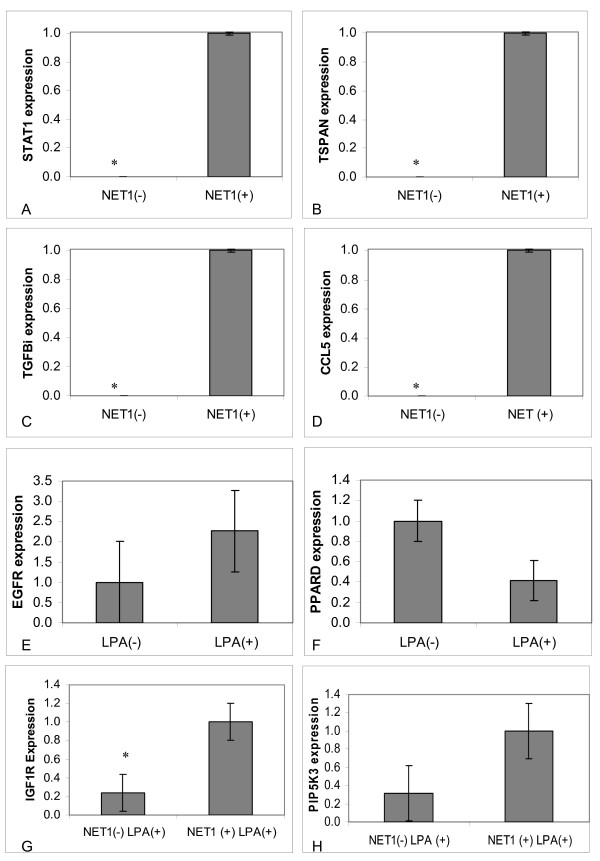
**Validation of gene array findings by real time PCR**. Figure 6A: STAT 1 mRNA expression in control cells transduced with non targeting shRNA (NT) [shown in all diagrams in Figure 6 as NET1 (+)] and knockdown cells transduced with NET1 targeting shRNA [shown in all diagrams in Figure 6 as NET1(-)] Figure 6B: TSPAN 1 expression in control cells transduced with non targeting shRNA (NT) cells and NET1 knockdown cells transduced with NET1 targeting shRNA. Figure 6C. TGFBi expression in control cells transduced with non targeting shRNA (NT) and NET1 knockdown cells transduced with NET1 targeting shRNA. p < 0.0001 Figure 6D. CCL5 mRNA expression in control cells transduced with non targeting shRNA (NT) and knockdown cells, p < 0.0001; Figure 6E. EGFR expression in untreated and LPA treated AGS cells. Figure 6F: PPARD expression in untreated and LPA treated AGS cells. Figure 6G: IGF1R expression in LPA treated cells tranduced with non targeting shRNA control cells and LPA treated NET1 knockdown cells transduced with NET1 targeting shRNA, p = 0.0001. Figure 6H. PIP5K3 expression in LPA treated cells tranduced with non targeting shRNA control cells and LPA treated NET1 knockdown cells transduced with NET1 targeting shRNA. P < 0.0001.

Several genes from each category were chosen for further analysis as shown in Figure 6. From the NET1 dependent upregulated genes, STAT1, TSPAN1, TGFβI and CCL5 were chosen. From the LPA dependent upregulated genes, EGFR and PPARδ were selected for further analysis. Finally IGF1R and PIP5K3 were chosen from the NET1 dependent LPA driven upregulated genes. Real time PCR was performed using gene specific primers. All observations made by gene array experiments were confirmed using real time PCR (Figure 6).

## Discussion

NET1 has been shown to be involved in the progression of GC [6]. NET1 is a known activator of Rho A, and its actions, are at least in part RhoA mediated. In this current study, a GC cell line with stable low levels of NET1 expression was created to further investigate its role in the disease process by assessing the functional and global transcriptomic effects of this knockdown.

Previous studies have shown that other guanine exchange factors, such as GEFT, promote cellular proliferation [26,27]. In this study proliferation was not affected when NET1 was suppressed, suggesting that it is not critical for the proliferation of GC cells. We have previously shown the proliferation of GC cells to be reduced 48 hr following transient transfection of NET1 targeting siRNA [6]. In this current study, the proliferation of cells in which NET1 had been stably reduced via lentiviral mediated transduction of NET1 targeting shRNA was assessed many cell passages later. These data illustrate that NET1 is not essential for AGS cell proliferation and the importance of establishing stable long term knockdown models in order to truly assess gene function.

Other guanine nucleotide exchange factors such as Tiam1 have been shown to increase migration and invasion of cancer cells, such as breast and colon cancer [28-30]. In our study, migration was assessed using wound healing assays. Significantly fewer NET1 knockdown cells migrated across the scratch when compared to wild type AGS cells and control non target cells (P < 0.05). This reduction in migration was not seen when the non target cells were compared to the wild type AGS cells, supporting the hypothesis that the motility of the cells was in part NET1 dependent. When invasion of these cells was compared, the NET1 knockdown cells displayed dramatically decreased invasion compared to control cells transduced with non targeting scrambled shRNA cells. The non target cells, which serve as a control for the transduction process, did not show a significant difference in invasion when compared to the AGS cells. These results further support a central role for NET1 in GC cell migration and invasion.

In this study, we successfully performed oligonucleotide microarrays on NET1 knockdown cells and control GC cells transduced with non targeting shRNA. We identified 3 specific GC related expression patterns - NET1 dependent gene changes, LPA driven gene changes, and NET1 dependent LPA driven events. We further identified the NET1 dependent pathways and cellular processes through identification of the functional classification of the differentially expressed genes. Our microarray analysis showed that gene families relating to cell motility, chemotaxis and cytoskeletal organisation were upregulated in the presence of high levels of NET1. This supports previous data showing NET1 to be involved in cytoskeletal reorganisation and cellular motility [7]. We have also demonstrated that genes involved in regulation of apoptosis, cell death and DNA repair are down-regulated in the presence of high levels of NET1. As these traits are vital to cellular proliferation in the cancer setting, these data support a role for NET1 as a suppressor of these pathways.

LPA is a potent signalling molecule, that plays a key role in driving angiogenesis and metastasis [31,32]. We have previously shown LPA to drive RhoA activation in GC, and furthermore, that NET1 mediates this process [7]. Furthermore, we have previously shown the functional effect of LPA in GC whereby treatment with LPA resulted in an increase in AGS GC cell invasion and migration [7]. In this study we included LPA stimulated control and NET1 knockdown cells in our gene array studies to identify what LPA driven gene changes are NET1 dependent. In this study, stimulation of AGS cells with LPA resulted in the differential expression of over 2000 genes, the majority of which were involved in cellular metabolic processes.

We shortlisted genes from each microarray comparison for quantitative Real Time PCR validation. This was done to ensure these genes were not falsely identified by gene array but also because they were related to cell functions in which NET1 is established e.g. chemotaxis, or they were related to GC, a disease wherein NET1 plays an important role. STAT1 (signal transducer and activator of transcription 1) was up-regulated in AGS cells with high levels of NET1 in comparison with NET1 knockdown cells. We shortlisted STAT1 as recent evidence has emerged supporting a role for it in gastric inflammation and tumorigenesis in mice [33-36]. In this study TSPAN1 mRNA expression levels correlated with NET1 with decreased expression in NET1 knockdown cells. TSPAN1 is upregulated in oesophageal, ovarian and endometrial cancers [37-39]. TSPAN1 is overexpressed in GC where it correlates negatively with the degree of tumour differentiation and survival, and positively with depth of invasion and lymph node involvement [40]. TGFBi inhibits cell adhesion and correlates with advanced metastasis in colorectal cancer [41]. We have shown a clear relationship between NET1 expression and TGFBi expression, with negligible TGFBi expression detected in NET1 knockdown cells. CCL5 (Chemokine ligand 5) expression levels have been shown to correlate with poor outcome in GC [42]. While its role in GC remains to be fully elucidated, we have shown that NET1 is important for expression of CCL5 in GC.

In this study, we have shown by microarray and RTPCR that EGFR, a cell surface receptor for members of the EGF family is up regulated upon LPA treatment. EGFR is known to be crucial for cancer cell survival and differentiation [43] and is therefore a target for cancer therapy [44,45] using EGFR inhibitors. In this study we shortlisted EGFR as LPA and EGFR interplay has recently been shown to promote GC cell motility and invasion [46]. In this study we have shown that PPARD expression was down regulated in AGS cells treated with LPA. PPARD has been shown to be associated with colonic tumorigenesis in mice [47]. PPARD expression has been described in squamous head and neck cancers [48] and in endometrial cancer [49]. LPA has been described as an activator of PPAR gamma, though no reports of LPA induction or inhibition of PPARD have yet been published.

IGF1R (insulin like growth factor receptor 1) is over expressed in malignant tissues and acts as an anti apoptotic agent enhancing cell survival [50,51]. IGF1R has been shown to be a predictor of poor outcome in patients with GC [52]. Our study has shown LPA mediated IGF1R expression was dependent on NET1 expression. Coronas et al 2008 have suggested a role for PIP5K3 (Phosphatidylinositol-3-phosphate/phosphatidylinositol 5-kinase, type III) in oncogenesis [53]. PIP5K3 has also been proposed as a mediator of EGFR transcription function in bladder carcinoma [54]. Its role in gastric oncogenesis is not yet clear, however our data shows that LPA induced PIP5K3 expression is NET1 dependent in GC.

## Conclusion

In summary, we have shown that NET1 is a potent mediator and an important player in GC cell invasion. Suppression of NET1 results in significantly reduced motility and invasion of AGS GC cells in vitro. These traits are essential for tumor metastasis, thereby supporting a role for NET1 in the disease setting. This is also the first report of the 'NET1 transcriptome', a gene expression signature of GC invasion. As well as providing a functional insight into the biology and signalling underpinning NET1-mediated cell invasion, this dataset may prove useful in the development of biomarkers for disease aggressiveness, or in providing targets for therapeutic intervention. Genes involved in cellular differentiation and proliferation, cell motility, and cell-cell adhesion are up-regulated when high levels of NET1 are present, indicating that NET1 drives these cellular processes that contribute to the aggressiveness of GC. The specific mechanisms require further investigation possibly across other cell line models of GC but may very well serve as a potential targets for GC therapies in the future.

GEO Microarray Data Accession: GSE26309

## Competing interests

The authors declare that they have no competing interests.

## Authors' contributions

All authors have read and approved the final manuscript. GB performed the majority of the lab work described herein and wrote the manuscript. DS contributed to the analysis of gene array data. PMcM advised on and ensured the clinical relevance of this study and contributed to the preparation and revision of this manuscript. PD contributed to overall experimental design and was involved in the revision of this manuscript. DM was involved with the design and day to day roll out of the laboratory programme described herein as well as being involved with the preparation and revision of this manuscript.

## Pre-publication history

The pre-publication history for this paper can be accessed here:

http://www.biomedcentral.com/1471-2407/11/50/prepub
